# Hepatic Growth Factor as a Potential Biomarker for Lung Adenocarcinoma: A Multimodal Study

**DOI:** 10.3390/cimb47030208

**Published:** 2025-03-19

**Authors:** Mengxuan Sun, Yang Yu, Hanci Zhu, Yan Yao, Xintong Zhou, Xue Wang, Yubao Zhang, Xiaowei Xu, Jing Zhuang, Changgang Sun

**Affiliations:** 1 College of First Clinical Medicine, Shandong University of Traditional Chinese Medicine, Jinan 250355, China; sunmengxuandoc@163.com (M.S.); 13105405091@163.com (H.Z.); 17865991065@163.com (Y.Y.); zxt199703@163.com (X.Z.); 15610562275@163.com (Y.Z.); 2 State Key Laboratory of Quality Research in Chinese Medicine, Faculty of Chinese Medicine, Macau University of Science and Technology, Avenida Wai Long, Taipa, Macau 999078, China; 3 College of Traditional Chinese Medicine, Shandong Second Medical University, Weifang 261000, China; 17861431558@163.com (X.W.); 18866778991@163.com (X.X.)

**Keywords:** inflammatory factors, lung adenocarcinoma, HGF, Mendelian randomization, transcriptome sequencing

## Abstract

(1) Background: Despite previous studies linking inflammatory cytokines to lung adenocarcinoma (LUAD), their causal mechanisms remain unclear. This study aims to explore the causal relationship between inflammatory cytokines and LUAD to fill this knowledge gap. (2) Methods: This study employs a comprehensive approach, integrating Mendelian randomization (MR) analysis, single-cell RNA sequencing (scRNA-seq), and transcriptomic sequencing (RNA-seq) data to investigate the relationship between inflammatory cytokines and LUAD. (3) Results: In forward MR analysis, elevated levels of hepatocyte growth factor (HGF), interleukin-1 receptor antagonist (IL-1RA), IL-5, monocyte chemoattractant protein-3, and monokine induced by interferon-γ were causally associated with an increased risk of LUAD. In reverse MR analysis, LUAD exhibited a positive causal relationship with the levels of regulated upon activation normal T cell expressed and secreted factor (RANTES) and stromal cell-derived factor-1α. The scRNA-seq data further identified specific cell populations that may influence LUAD onset and progression through the expression of particular inflammatory genes and intercellular communication. RNA-seq data analysis highlighted the role of the HGF gene in LUAD diagnosis, demonstrating its strong correlation with patient prognosis and immune cell infiltration within the tumor microenvironment. (4) Conclusions: The findings reveal a causal relationship between inflammatory cytokines and LUAD, with HGF emerging as a potential biomarker of significant clinical relevance. This study provides new insights into the molecular mechanisms underlying LUAD and lays the foundation for future therapeutic strategies.

## 1. Introduction

Lung adenocarcinoma (LUAD) is one of the most aggressive tumor types, with an overall survival (OS) rate of less than five years [1]. Factors such as chemotherapy resistance and evasion of apoptosis often negatively impact the effectiveness of antitumor therapy in LUAD patients, leading to tumor recurrence and poor prognosis [2,3]. Therefore, the development of novel biomarkers and therapeutic strategies is crucial for improving early detection, optimizing treatment regimens, and enhancing prognostic assessment. Epidemiological studies, histopathological analyses, and functional research have confirmed that inflammatory cytokines play a pivotal role in cancer initiation, progression, and treatment [4,5,6,7]. Various cell types produce inflammatory mediators and secrete them into circulation, which may induce tissue damage, epithelial cell mutations, endothelial dysfunction, angiogenesis, immunosuppression, and stromal remodeling, ultimately promoting cancer development [8]. Additionally, inflammatory cells can release factors that activate key inflammatory signaling pathways in tumor cells, thereby driving tumor progression and metastasis. On the other hand, inflammatory cytokines exhibit diverse applications and potential value in cancer therapy. Cytokine-based therapies, such as interferon-α, granulocyte-macrophage colony-stimulating factor, interleukin-2 (IL-2), and IL-12, exert antitumor effects by directly leveraging specific cytokines [9]. Meanwhile, anti-cytokine therapies, such as infliximab, which blocks tumor necrosis factor (TNF), or siltuximab, which targets IL-6, intervene in tumor progression by inhibiting pro-tumorigenic cytokines [10,11]. Despite extensive clinical research on the interplay between inflammatory cytokines and LUAD, it remains controversial whether these factors contribute to LUAD initiation or merely influence its progression. This study integrates Mendelian randomization (MR) analysis with single-cell RNA sequencing (scRNA-seq) and transcriptomic sequencing (RNA-seq) approaches to elucidate the roles of specific inflammatory cytokines and their associated genes in LUAD.

## 2. Materials and Methods

### 2.1. Two-Sample Bidirectional MR Analysis

For the MR analysis, genetic variants were selected from the genome-wide association study (GWAS) database (https://www.ebi.ac.uk/gwas/, accessed on 25 June 2024) [12], using single nucleotide polymorphisms (SNPs) as instrumental variables (IVs) to assess causal relationships. Data on 41 inflammatory cytokines were obtained from 8293 Finnish individuals, while LUAD data were collected from 18,336 European patients (Supplementary Table S1). SNPs associated with inflammatory cytokines and LUAD were identified based on genome-wide significance (*p* < 5 × 10^−6^) and independence criteria (r^2^ = 0.001, kb = 5000). An F-statistic greater than 10 confirmed a robust association between IVs and exposure factors. MR analysis was performed using the “TwoSampleMR” R Package (version 0.6.3) and ‘MRInstruments’ R package (version 0.3.2) from the MR-Base platform [13]. The leave-one-out analysis and Cochran’s Q test were used to evaluate SNP heterogeneity, while MR-PRESSO and MR-Egger intercept analysis were applied to detect pleiotropy-induced bias.

### 2.2. Single-Cell RNA Sequencing (scRNA-Seq) Analysis

The scRNA-seq dataset GSE223923 was retrieved from the Gene Expression Omnibus (GEO, https://www.ncbi.nlm.nih.gov/geo/, accessed on 29 June 2024) [14], which includes four LUAD samples. Data preprocessing was conducted using the “Seurat” package (version 5.1.0), with stringent quality control applied to filter out low-quality cells. A doublet detection procedure was implemented, followed by standardization, feature selection, and normalization of the data. Principal component analysis was performed to identify key sources of variation. The harmony algorithm was employed to integrate data and eliminate batch effects. Dimensionality reduction and clustering were conducted using the uniform manifold approximation and projection (umap) algorithm to define cell subpopulations. Cell type annotation was performed using the “SingleR” tool (version 2.4.1) based on cell type-specific gene sets [15]. The expression of the seven inflammatory cytokines identified as positive results in the MR analysis was evaluated across different cell clusters. Cell–cell communication networks and related signaling pathways were analyzed and visualized using the “CellChat” tool (version 1.6.1), which assesses ligand–receptor interactions among different cell types, revealing associations between specific signaling pathways and cellular subpopulations. Additionally, gene ontology (GO) and Kyoto Encyclopedia of Genes and Genomes (KEGG) pathway analyses were conducted to evaluate the biological functions and signaling pathways of the identified inflammatory cytokine-associated genes.

### 2.3. RNA-Seq Analysis

RNA-seq data were obtained from the GEO database, specifically from the GSE14814, GSE19188, and GSE68571 datasets, comprising high-throughput sequencing data from 197 LUAD samples and 65 normal lung tissue samples [16,17,18]. A random forest (RF) algorithm, a machine learning method, was applied to screen the seven genes that yielded positive results in the MR analysis, identifying the core genes among them. A boxplot was used to visualize the differential expression levels of these core genes between normal and LUAD tissues. To assess the diagnostic predictive accuracy of the core genes, receiver operating characteristic (ROC) curves were generated using the “PROC” package (version 1.18.5), and the area under the curve (AUC) was calculated. Based on the median gene expression levels, samples were categorized into high- and low-expression groups, followed by Kaplan–Meier (K–M) survival analysis to compare survival curves between normal and LUAD groups. The log-rank test was used to evaluate the statistical significance of core gene expression in survival outcomes. Key genes with significant AUC values and survival curve statistics were selected for further immune infiltration analysis. Subsequently, diagnostic AUC validation was performed using the external dataset GSE31210 [19]. Finally, the “CIBERSORT” (version 0.1.0) algorithm was employed to categorize samples into high- and low-expression groups based on the median expression levels of key genes. The relative abundance differences of various immune cell types in these expression groups were comprehensively assessed to elucidate the distribution characteristics of immune cells within the tumor microenvironment.

## 3. Results

### 3.1. Bidirectional Causal Effects of Inflammatory Cytokines on LUAD

**Figure 1:**
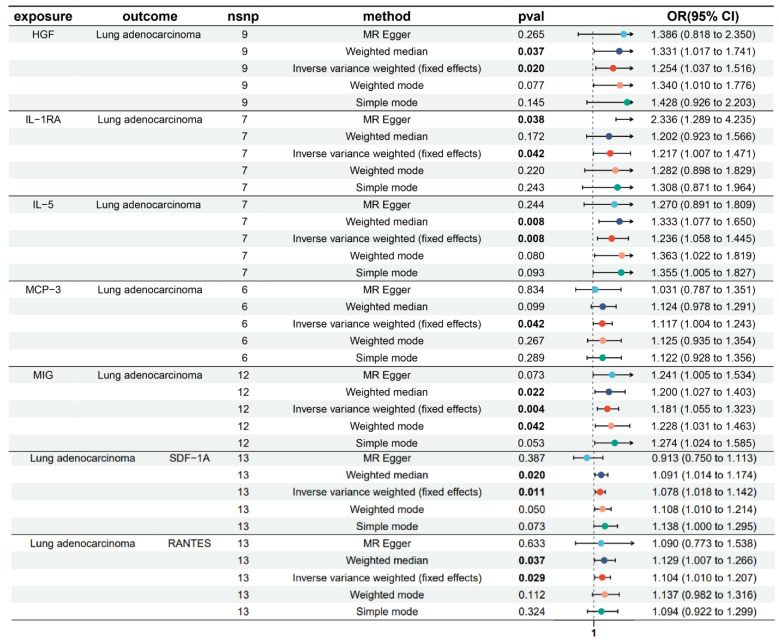
Bidirectional Mendelian randomization analysis of inflammatory cytokines and lung adenocarcinoma.

During the selection of IVs, forward MR analysis identified a causal relationship between LUAD and SNPs associated with hepatocyte growth factor (HGF), interleukin-1 receptor antagonist (IL-1RA), IL-5, monocyte chemoattractant protein-3 (MCP-3), and monokine induced by interferon-γ (MIG). The number of corresponding SNPs for each cytokine was 9, 7, 7, 6, and 12, respectively. In reverse MR Analysis, 13 SNPS in normal T cell expressed and secreted factor (RANTES) and stromal cell-derived factor-1α (SDF-1A) were each found to be causally associated with LUAD (Supplementary Table S2). Inverse variance-weighted (IVW) analysis demonstrated significant positive causal associations between LUAD and the inflammatory cytokines HGF (IVW-OR: 1.254, *p* = 0.020), IL-1RA (IVW-OR: 1.217, *p* = 0.042), IL-5 (IVW-OR: 1.236, *p* = 0.008), MCP-3 (IVW-OR: 1.117, *p* = 0.042), and MIG (IVW-OR: 1.181, *p* = 0.004). Meanwhile, in the reverse MR analysis, LUAD was found to have a causal relationship with elevated levels of RANTES and SDF-1A, with IVW results showing an odds ratio of 1.104 (*p* = 0.029) for RANTES and 1.078 (*p* = 0.011) for SDF-1A (Figure 1).

### 3.2. MR Sensitivity Analysis

The MR sensitivity analysis revealed that the *p*-values for both the MR-Egger intercept test and the MR-PRESSO test were greater than 0.05, indicating the absence of pleiotropy. Additionally, Cochran’s Q test and leave-one-out sensitivity analysis further confirmed the stability of the causal relationship (Supplementary Table S3, Supplementary Figures S1–S7). Although some inflammatory cytokines did not reach statistical significance in other MR methods, the consistent trend observed in the analyses suggests that these cytokines may increase the genetic predisposition to LUAD (Supplementary Table S4).

### 3.3. scRNA-Seq Cell Clustering and Gene Expression Profiles

**Figure 2:**
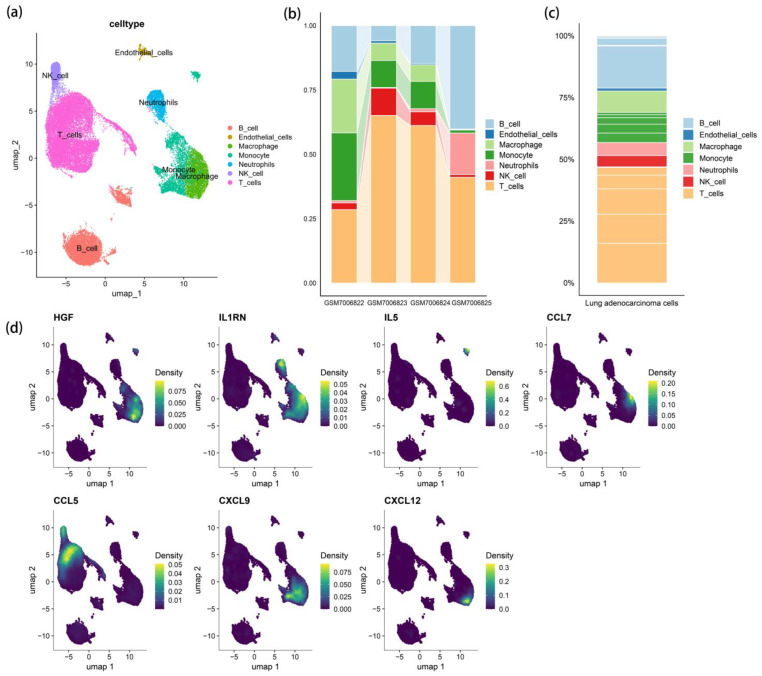
(**a**) LUAD cell cluster map; (**b**) Cell population distribution in a single sample; (**c**) Distribution of all sample cell populations; (**d**) Distribution of inflammatory genes among cell populations.

During the study, seven major cell populations were identified through automated annotation and marker gene-based classification. These included monocytes, macrophages, neutrophils, T cells, natural killer (NK) cells, B cells, and endothelial cells. Based on the results of MR analysis, seven key genes closely associated with inflammatory responses—HGF, IL1RN (also known as IL1RA), IL5, chemokine (C-C motif) ligand 7 (CCL7, also known as MCP3), CCL5 (also known as RANTES), chemokine (C-X-C motif) ligand 9 (CXCL9, also known as MIG), and CXCL12 (also known as SDF-1)—were selected for further analysis of their differential expression patterns across these seven cell populations (Figure 2). The results indicated that HGF, CCL7, CXCL9, and CXCL12 were highly expressed in macrophages, IL1RN was predominantly distributed in monocytes, macrophages, and neutrophils, IL5 was mainly expressed in monocytes, and CCL5 was primarily present in T cells and NK cells. The differential expression of these genes among various cell populations may play a crucial role in modulating inflammatory responses and related pathophysiological processes within the tumor microenvironment.

### 3.4. Receptor–Ligand Mediated Cell Communication

**Figure 3:**
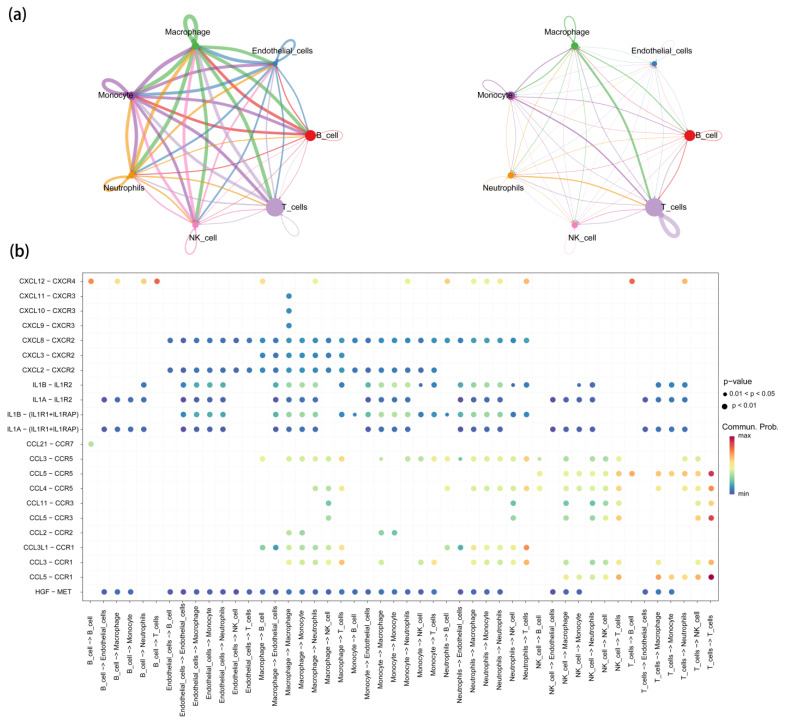
(**a**) The frequency and intensity of intercellular communication; (**b**) Bubble plot of receptor–ligand-mediated intercellular communication.

The scRNA-seq analysis clearly revealed the intricate intercellular interactions and communication networks in LUAD. A detailed investigation of these networks indicated that T cells ranked highest in both the frequency and intensity of intercellular communication. Notably, interactions between T cells, neutrophils, macrophages, and monocytes exhibited the strongest signaling intensity within the entire communication network (Figure 3). Additionally, several receptor–ligand pairs that were significantly associated with the MR analysis results were found to mediate signal transmission within specific cellular pathways. For instance, HGF–mesenchymal-epithelial transition factor (MET), CCL5–chemokine (C-C motif) receptor 1 (CCR1), CCL5–CCR5, CCL5–CCR3, CXCL9–CXCR3, CXCL12–CXCR4, IL1A–IL1R2, and IL1β (B)–IL1R2 were highly expressed in intercellular communication (*p* < 0.01) (Supplementary Figure S8). Of particular interest, as shown in Figure 3, HGF has only one receptor, MET, and the HGF–MET receptor–ligand pair plays a crucial role in multiple intercellular interactions across the majority of cell populations. This suggests that HGF–MET may serve as a key regulatory axis influencing cellular behavior and function (Figure 3).

**Figure 4:**
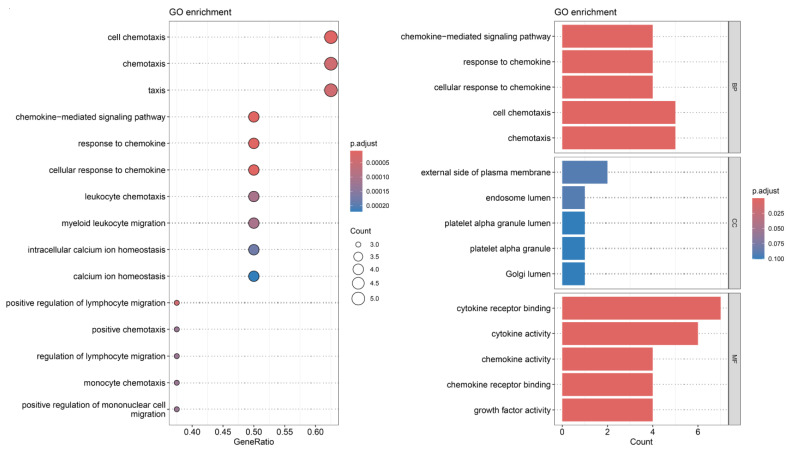
GO function and KEGG pathway annotation diagram.

The biological process (BP) enrichment analysis demonstrated that inflammatory cytokines are extensively involved in multiple biological processes, with pathways related to chemokine-mediated signaling being particularly prominent. The molecular function (MF) analysis revealed that at the molecular level, inflammatory cytokines are primarily associated with cytokine activity and cytokine receptor binding. The cellular component (CC) enrichment analysis further highlighted the activity of inflammatory cytokines in specific cellular structures, such as the extracellular region of the plasma membrane and the lumen of platelet alpha granules. Furthermore, the KEGG analysis identified key pathways related to inflammatory cytokines, including cell chemotaxis and calcium homeostasis (Figure 4).

### 3.5. Identification of HGF in the LUAD Transcriptome

**Figure 5:**
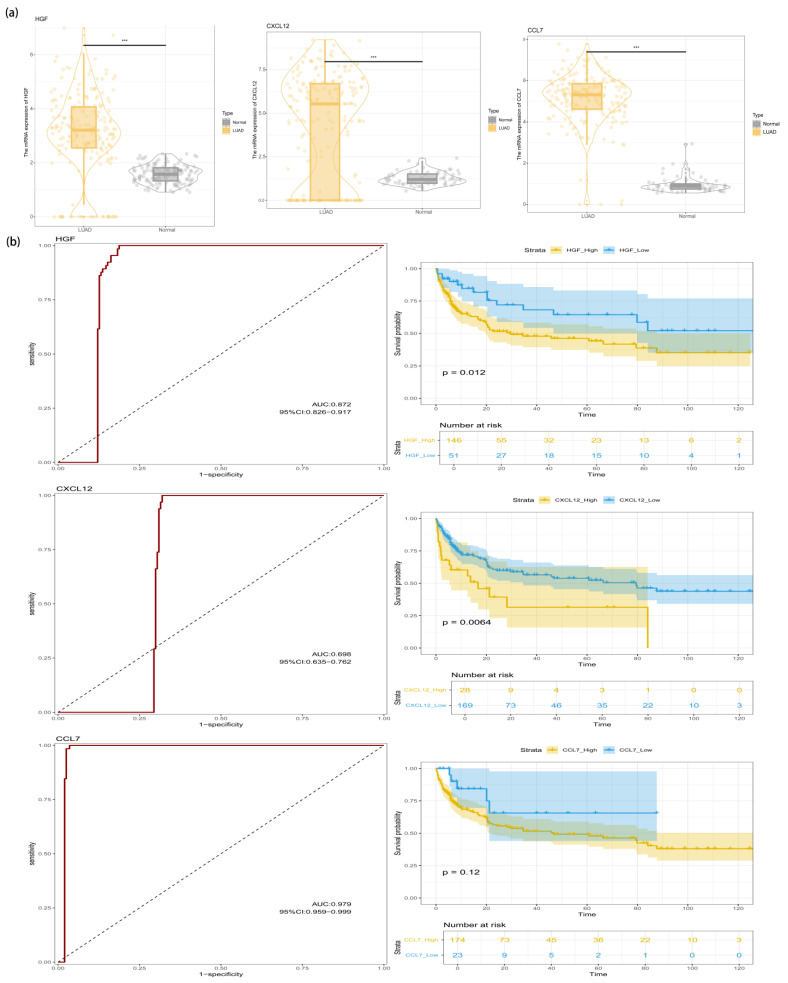
(**a**) Differential expression of HGF, CXCL12, and CCL7 between normal and LUAD tissues; (**b**) ROC curve analysis and K–M survival curves of HGF, CXCL12, and CCL7. (*** *p* < 0.001).

Although we have observed the expression profiles and intercellular communication patterns of inflammatory factors in distinct LUAD cell clusters, the overall expression levels of these inflammatory genes in LUAD require further investigation. To address this, an RF algorithm—a machine learning method—was employed to screen seven inflammatory genes, ultimately identifying HGF, CXCL12, and CCL7 as the core genes for further analysis (Supplementary Figure S9). Comparative expression analysis between normal lung tissue and LUAD tissue revealed significant differences in the expression levels of these three genes. To evaluate the diagnostic accuracy of HGF in LUAD, we conducted an ROC curve analysis. The results demonstrated that the AUC values for HGF, CXCL12, and CCL7 were 0.872, 0.698, and 0.979, respectively (Figure 5). Subsequently, patients were stratified into high-expression and low-expression groups based on the median expression levels of these genes. K–M survival curve analysis indicated that the expression levels of HGF and CXCL12 were significantly correlated with OS (*p* < 0.05). Integrating the diagnostic AUC results and prognostic K–M survival analysis, we identified HGF as a promising biomarker with both diagnostic and prognostic potential in LUAD. Furthermore, its diagnostic value was validated in an independent external dataset, yielding an AUC of 0.858 (Supplementary Figure S10).

### 3.6. Immune Landscape Analysis

**Figure 6:**
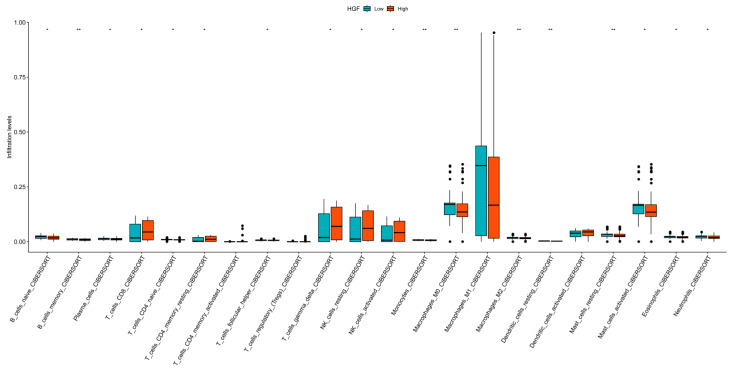
Immune infiltration differences between high and low HGF expression groups. (* *p* < 0.05, ** *p* < 0.01).

Analysis of scRNA-seq data revealed widespread distribution of inflammatory cytokines across immune cell populations, with HGF being predominantly expressed in monocytes and macrophages. To further explore the immune infiltration landscape of HGF in high-throughput transcriptomic data, samples were classified into high-expression and low-expression groups based on the median HGF expression level. The analysis demonstrated significant differences in immune infiltration between these groups across multiple immune cell subsets, particularly in memory B cells, monocytes, M0 and M2 macrophages, resting dendritic cells, and resting mast cells. Notably, M1 macrophages exhibited extensive infiltration in both the high- and low-expression groups (Figure 6). Considering the findings from single-cell analysis, there is strong evidence supporting a close association between HGF and macrophage populations. 

## 4. Discussion

Extensive fundamental, clinical, and epidemiological studies have demonstrated that inflammation plays a crucial regulatory role in cancer initiation, progression, and response to therapy [20]. During tumor initiation, inflammation fosters tumorigenesis by promoting cell proliferation, inhibiting apoptosis, and regulating genomic stability. As the tumor progresses, inflammation drives its advancement through mechanisms such as epigenetic modifications, aberrant gene expression, vascular abnormalities, and tumor neovascularization [21]. Cytokines, as hallmark mediators of chronic inflammation and autoinflammatory diseases, serve as key bridges linking inflammation and cancer [22,23]. Tumor cells exploit cytokines and their receptors to stimulate immune responses and promote angiogenesis, thereby facilitating tumor growth and metastasis. Meanwhile, dysregulation of inflammatory cytokines acts as a catalyst in malignancy progression, significantly contributing to tumor deterioration. In the pathogenesis of LUAD, cytokine dysregulation plays a central role, exerting multifaceted effects on tumor initiation, progression, and prognosis through intricate signaling pathways [24,25,26]. Previous studies have confirmed that inhibiting key cytokine signaling pathways effectively suppresses tumor growth and metastasis [20,24].

The onset and progression of LUAD are closely intertwined with various inflammatory cytokines and inflammatory responses. In LUAD patients, inflammatory responses are often accompanied by abnormal expression of multiple cytokines. Numerous studies have reported significant associations between the expression levels of inflammatory cytokines—including IL-6, CXCL9, TNF-α, IL-17A, and IL-32—and LUAD tumor stage, metastasis, and prognosis [27,28,29,30,31,32]. These cytokines are primarily secreted by T cells and monocytes/macrophages, triggering a cascade of downstream effects [33]. Their primary mechanisms of action include promoting angiogenesis in the innate immune response to natural inflammation, increasing DNA adduct levels, and inducing DNA damage through the synthesis of reactive substances [34]. Thus, inflammatory cytokines hold great potential as therapeutic targets for LUAD prevention and treatment, providing a solid foundation for developing more personalized and effective therapeutic strategies.

To elucidate the intricate interplay between specific inflammatory cytokines and precision therapy for LUAD, this study integrates GWAS data from individuals of European ancestry, single-cell sequencing data, and transcriptomic data, conducting an independent multi-modal analysis. The results of forward MR analysis showed that the level changes of HGF, IL-1RA, IL-5, MCP-3, and MIG were positively causally related to the occurrence and development of LUAD. The results of reverse MR analysis indicated that there was also a positive causal association between LUAD and RANTES and SDF-1A. Subsequently, leveraging single-cell transcriptomic data, this study identified cell type-specific gene expression patterns in LUAD, unveiling the heterogeneous expression of relevant inflammatory factors across distinct cellular populations within the tumor. Furthermore, an in-depth examination of ligand–receptor pair-mediated intercellular communication was conducted. Transcriptomic data analysis further highlighted the multifaceted role of the HGF gene in LUAD. HGF not only serves as a potential biomarker for LUAD diagnosis, but its expression levels also exhibit a strong correlation with patient survival outcomes. Additionally, the dynamic changes in HGF expression are closely linked to the composition and activation states of immune cells within the LUAD tumor microenvironment, potentially exerting a critical influence on immune evasion mechanisms and tumor response to therapy.

The aforementioned findings provide a preliminary macroscopic overview of the relationship between inflammatory cytokines and LUAD. Building upon this foundation, it is crucial to further dissect the specific impact of each inflammatory cytokine on LUAD initiation and progression to comprehensively elucidate their complex biological interplay. Specifically, IL-1RA, an effective antagonist of IL-1, competitively binds to the IL-1 receptor, thereby inhibiting the pro-inflammatory effects of IL-1β and modulating inflammation and tumor activation processes [35]. Studies have shown that IL-1RA expression is elevated in lung cancer tissues compared to normal tissues, and its increased levels are associated with improved progression-free survival (PFS) in patients [36,37]. Although IL-1RA possesses anti-inflammatory properties and theoretically may help suppress tumor progression, it could also exert an inhibitory effect on the body’s antitumor immune response [38].

IL-5, secreted by T cells, plays a regulatory role in the production, activation, aggregation, and recruitment of eosinophils within the tumor microenvironment [39]. A clinical observational study demonstrated a significant correlation between IL-5 levels in pleural effusions of LUAD patients and the number of malignant cells present [40]. Additionally, preoperative blood samples from non-small cell lung cancer (NSCLC) patients exhibited significantly elevated IL-5 levels, which markedly decreased following tumor resection [40].

MIG plays a complex role in tumor progression. Through signaling pathways such as Janus kinase/signal transducer and activator of transcription and nuclear factor kappa-light-chain-enhancer of activated B cells, MIG mediates responses to various cytokines, thereby influencing the tumor microenvironment [41]. In LUAD patients, MIG expression is affected by CTNNA2 gene mutations and is closely associated with improved PFS and OS [29,42]. Furthermore, serum MIG levels in NSCLC patients were significantly higher preoperatively than postoperatively [43].

In LUAD, high MCP-3 expression is associated with an increase in tumor-associated macrophages, a phenomenon that may enhance vascular permeability and thereby promote tumor progression [44]. In-depth studies have shown that the expression of MCP-3 is regulated by the transcription factor spleen focus forming virus proviral integration oncogene 1 and is directly related to the accumulation of M2 macrophages and the malignant progression of LUAD cells [44].

In LUAD, elevated expression of RANTES is associated with poor prognosis. It may regulate the tumor microenvironment by affecting the function of regulatory T cells and CD8+ T cells [45,46]. Although RANTES expression seems to correlate with better survival outcomes in early-stage LUAD patients, its role becomes more complex during the entire course of disease progression [47].

SDF-1 and its receptors, CXCR4 and CXCR7, can promote tumor cell proliferation, survival, angiogenesis, and metastasis [48,49]. In NSCLC, cancer-associated fibroblasts produce SDF-1, which, upon binding to CXCR4, enhances the epithelial–mesenchymal transition and invasion capacity of cancer cells [50]. Notably, silencing CXCR7 not only reverses the resistance of epidermal growth factor receptor (EGFR)-mutant NSCLC cells to EGFR tyrosine kinase inhibitors (TKIs) but also promotes the transition of cancer cells to an epithelial phenotype [51].

Interestingly, based on the results of MR studies, we further conducted single-cell transcriptomic and ranscriptomic analyses on inflammatory factors causally related to LUAD. After integrating all the analyses, this study found that HGF, as a potential diagnostic biomarker for LUAD, has significant advantages. It is closely related to patient survival and is associated with immune cell infiltration patterns that likely influence the tumor’s immune evasion mechanisms and its response to treatment.

HGF, also known as scatter factor, is the natural ligand of the MET receptor tyrosine kinase, playing a central role in promoting cell motility, invasion, and morphogenesis [52]. From a biological mechanism perspective, the HGF/MET signaling axis can activate various downstream signaling pathways, such as rat sarcoma virus/rapidly accelerated fibrosarcoma, mitogen-activated protein kinase kinase/extracellular signal-regulated kinase, phosphatidylinositol 3-kinase/protein kinase B, and wingless-related integration site/β-catenin pathways, thereby participating in key growth processes such as tumor cell proliferation, survival, invasion, migration, and drug resistance [53]. In various cancers, abnormal activation of the HGF/MET signaling pathway is caused by multiple mechanisms, including gene amplification, overexpression, the presence of different functional gain-of-function mutations, and abnormal secretion of HGF through autocrine or paracrine pathways [54].

Of particular importance, as a candidate mediator of TKI resistance, the increased expression of HGF can lead to activation and amplification of the c-MET receptor in cancer cells and tumor-associated stroma, which is closely related to the acquired resistance to EGFR-TKIs [55,56,57]. Peng et al. studied EGFR-TKI-resistant NSCLC cells with c-MET amplification, HGF, and EGFR-T790M, three known resistance mechanisms, and found that HGF, MET amplification, and EGFR-T790M upregulate the expression of programmed cell death ligand 1 (PD-L1) in NSCLC and assist tumor cells in immune evasion through different mechanisms [58]. Further in-depth exploration revealed that macrophages play a crucial role in the complex process of immune evasion. Hepatocyte growth factor is a key regulatory factor in the polarization of macrophages toward the M2 phenotype. When HGF levels increase in the tumor microenvironment, tumor-infiltrating macrophages are more likely to transition into the immunosuppressive M2-polarized phenotype [59,60]. M2-type tumor-associated macrophages highly express interleukin-10 and transforming growth factor-β, both of which are associated with tumor-promoting effects. This weakens the body’s antitumor immune response, leading to a reduced therapeutic response to immune checkpoint inhibitors [59,61]. Studies have shown that targeting HGF can restore T-cell activity and exert a synergistic antitumor effect when combined with immune checkpoint inhibitors [62,63].

In addition to its critical role in immune regulation and its close association with tumor therapy, HGF also has a significant impact on tumor cell drug resistance and clinical phenotypic manifestations. In LUAD cells with EGFR mutations, overexpression of HGF significantly induces resistance to the first-generation EGFR-TKI drug gefitinib [64]. Clinical evidence shows that HGF expression levels are often elevated in NSCLC patients, especially in those with relapse [65,66,67,68]. Additionally, one-third of EGFR-mutant patients who are unresponsive to EGFR-TKIs exhibit high levels of HGF expression in their tumors [69]. Related to this study, commonly used anticancer drugs such as erlotinib, crizotinib, and bevacizumab, when used in combination, can inhibit the growth of EGFR-TKI-resistant tumors triggered by HGF in EGFR-mutant tumors [57]. Onartuzumab, a MET monoclonal antibody, can block HGF-induced MET activation, and its combination with erlotinib may benefit EGFR-mutant NSCLC patients with high HGF expression [70]. Therefore, inhibiting HGF overexpression and affecting MET activation appears to be a reliable approach to intervening in the treatment resistance of LUAD and suppressing tumor growth.

Given the aberrant expression of HGF in the clinical context of lung cancer and its critical impact on specific treatments, exploring its potential applications in lung adenocarcinoma diagnosis and treatment is of great significance. First, in detecting lung adenocarcinoma patients, HGF protein expression levels can be localized in tissue samples through immunohistochemistry to assess its paracrine activity within the tumor microenvironment. Meanwhile, highly sensitive enzyme-linked immunosorbent assay techniques can quantitatively measure HGF concentrations in liquid samples such as blood or pleural effusion to aid in diagnosis [71]. Additionally, quantitative polymerase chain reaction or next-generation sequencing can be employed to analyze HGF gene amplification or transcriptional abnormalities, while immunoblotting or mass spectrometry can further detect downstream signaling pathways of HGF, such as the phosphorylation status of the MET receptor [72,73]. These multidimensional detection methods provide strong support for the clinical application of HGF. Although HGF holds substantial clinical value as a biomarker and can be translated into clinical practice through multiple approaches, challenges remain regarding standardization of its detection. Standardizing HGF measurements across different clinical settings, such as between tissue and blood samples, faces several obstacles. The complexity of tissue samples, fluctuations in HGF concentrations in blood samples, and variations in sensitivity among different detection techniques may all affect measurement accuracy. Furthermore, standardization is needed for gene detection and signaling pathway analysis; dynamic monitoring of HGF level fluctuations and associated molecular characteristics before and after treatment is essential for optimizing therapeutic strategies, necessitating standardized monitoring and analytical protocols. Conducting clinical trials on HGF-targeted combination therapies also requires standardized criteria for patient selection, treatment regimens, and efficacy evaluation. Addressing these standardization challenges is crucial for improving the clinical translation of HGF, achieving precision medicine, and enhancing survival and prognosis in lung adenocarcinoma patients. Therefore, further investigation into these challenges is necessary to identify potential solutions and strategies for improving the accuracy and reliability of HGF detection.

In conclusion, this study established a multidimensional analysis framework to explore the causal relationship between inflammatory factors and LUAD. By combining MR analysis with single-cell sequencing data and transcriptomic data, this study identified inflammatory factor biomarkers associated with LUAD. These findings not only provide a new perspective for understanding the molecular mechanisms of LUAD but also guide subsequent research, especially in developing targeted therapeutic strategies for inflammatory pathways and disease prevention. However, this study has certain limitations. In terms of sample selection, all participants in the GWAS were of European ancestry. While this was intended to minimize demographic bias, significant genetic background and environmental differences across populations may affect the generalizability of the findings. Future studies should replicate these findings in non-European cohorts and evaluate HGF expression based on genetic characteristics specific to different ethnic groups to validate its generalizability. Regarding sample sources, existing single-cell transcriptomic and transcriptomic data are predominantly derived from untreated postoperative LUAD patients. Given the significant impact of chemotherapy, immunotherapy, and targeted therapy on patient outcomes, further validation in treated patient cohorts is necessary to ensure the clinical accuracy and reliability of the findings. At the mechanistic level, current research on the role of HGF in key processes such as immune evasion in LUAD remains insufficient. Future studies could establish HGF knockout models in LUAD cell lines to investigate its effects on cellular behavior. Additionally, MET inhibition experiments could be conducted to suppress MET receptor activity and assess its impact on the HGF signaling pathway and immune evasion-related markers, thereby elucidating the mechanism by which HGF contributes to immune evasion. Furthermore, the therapeutic potential of HGF as a target for LUAD treatment remains underexplored. A comprehensive feasibility assessment is needed, including the design and implementation of multicenter clinical studies incorporating cases from various regional medical centers. Such studies would facilitate the analysis of associations between HGF-related biomarkers and therapeutic efficacy, ultimately advancing LUAD diagnosis and treatment research.

## 5. Conclusions

This study elucidates the critical roles of specific inflammatory factors and associated genes in the progression of LUAD through the integration of MR and transcriptomic analysis. HGF emerges as a key biomarker closely linked to disease diagnosis and patient prognosis.

## Data Availability

The original contributions presented in this study are included in the article/Supplementary Material. Further inquiries can be directed to the corresponding authors.

## Supplementary Materials

The following supporting information can be downloaded at: https://www.mdpi.com/article/10.3390/cimb47030208/s1.

## Abbreviations

The following abbreviations are used in this manuscript:

LUAD	lung adenocarcinoma
MR	Mendelian randomization
HGF	hepatocyte growth factor
MCP-3	monocyte chemoattractant protein-3
MIG	monokine induced by interferon-γ
RANTES	regulated upon activation normal T-cell expressed and secreted
SDF-1A	stromal cell-derived factor-1α
OS	overall survival
IL-2	interleukin-2
TNF	tumor necrosis factor
GWAS	genome-wide association study
SNPs	single nucleotide polymorphisms
IVs	instrumental variables
scRNA-seq	Single-Cell RNA Sequencing
umap	uniform manifold approximation and projection
GO	gene ontology
KEGG	Kyoto Encyclopedia of Genes and Genomes
RF	random forest
ROC	receiver operating characteristic
AUC	area under the curve
K-M	Kaplan-Meier
IL-1RA	interleukin-1 receptor antagonist
IVW	Inverse variance-weighted
NK	natural killer
CCL7	chemokine (C-C motif) ligand 7
CXCL9	chemokine (C-X-C motif) ligand 9
BP	biological process
MF	molecular function
CC	cellular component
PFS	progression-free survival
NSCLC	non-small cell lung cancer
EGFR	epidermal growth factor receptor
TKIs	tyrosine kinase inhibitors
PD-L1	programmed cell death ligand 1
