# Supplementary material for: Hepatic Growth Factor as a Potential Biomarker for Lung Adenocarcinoma: A Multimodal Study
Source: Curr Issues Mol Biol. 2025 Mar 19;47(3):208. doi: 10.3390/cimb47030208 (PMC11941628; doi:10.3390/cimb47030208)
Supplement: Supplementary file 1 [file cimb-47-00208-s001.zip › Supplementary Figures Legends.pdf]

Supplementary Figure Legends

Supplementary Figure S1. Main graphical results of MR between HGF and lung adenocarcinoma. (a) forest plots; (b) funnel plots; (c) scatter plots; (d) leave-one-out plots. MR: Mendelian randomization; SNP: single nucleotide polymorphism; HGF: hepatocyte growth factor.

Supplementary Figure S2. Main graphical results of MR between IL-1RA and lung adenocarcinoma. (a) forest plots; (b) funnel plots; (c) scatter plots; (d) leave-one-out plots. MR: Mendelian randomization; SNP: single nucleotide polymorphism; IL-1RA: interleukin-1 receptor antagonist.

Supplementary Figure S3. Main graphical results of MR between IL-5 and lung adenocarcinoma. (a) forest plots; (b) funnel plots; (c) scatter plots; (d) leave-one-out plots. MR: Mendelian randomization; SNP: single nucleotide polymorphism; IL-5: interleukin-5.

Supplementary Figure S4. Main graphical results of MR between MCP-3 and lung adenocarcinoma. (a) forest plots; (b) funnel plots; (c) scatter plots; (d) leave-one-out plots. MR: Mendelian randomization; SNP: single nucleotide polymorphism; MCP-3: monocyte chemotactic protein-3.

Supplementary Figure S5. Main graphical results of MR between MIG and lung adenocarcinoma. (a) forest plots; (b) funnel plots; (c) scatter plots; (d) leave-one-out plots. MR: Mendelian randomization; SNP: single nucleotide polymorphism; MIG: monokine induced by interferon-gamma.

Supplementary Figure S6. Main graphical results of MR between RANTES and lung adenocarcinoma. (a) forest plots; (b) funnel plots; (c) scatter plots; (d) leave-one-out plots. MR: Mendelian randomization; SNP: single nucleotide polymorphism;

RANTES: Regulated on Activation, Normal T cell Expressed and Secreted factor.

Supplementary Figure S7. Main graphical results of MR between SDF-1A and lung adenocarcinoma. (a) forest plots; (b) funnel plots; (c) scatter plots; (d) leave-one-out plots. MR: Mendelian randomization; SNP: single nucleotide polymorphism; SDF-1A: Stromal cell-derived factor-1α.

Supplementary Figure S8. Hierarchy diagram of cell-cell communication mediated by signaling pathways. (a) CCL signaling pathway network; (b) CXCL signaling pathway network; (c) HGF signaling pathway network; (d) IL1 signaling pathway network. CCL: C-C motif chemokine ligand; CXCL: C-X-C motif chemokine ligand; HGF: hepatocyte growth factor; IL1: interleukin 1.

Supplementary Figure S9. Random forest (RF) algorithm map of 7 inflammatory genes. HGF: hepatocyte growth factor; IL1RN: interleukin 1 receptor antagonist; IL5: interleukin 5; CCL7: C-C motif chemokine ligand 7; CCL5: C-C motif chemokine ligand 5; CXCL9: C-X-C motif chemokine ligand 9; CXCL12: C-X-C motif chemokine ligand 12.

Supplementary Figure S9. Validation of the AUC value of HGF in dataset GSE31210. AUC: area under the curve; HGF: hepatocyte growth factor.
