# Supplementary figures and images for: Hepatic Growth Factor as a Potential Biomarker for Lung Adenocarcinoma: A Multimodal Study
Source: Curr Issues Mol Biol. 2025 Mar 19;47(3):208. doi: 10.3390/cimb47030208 (PMC11941628; doi:10.3390/cimb47030208)

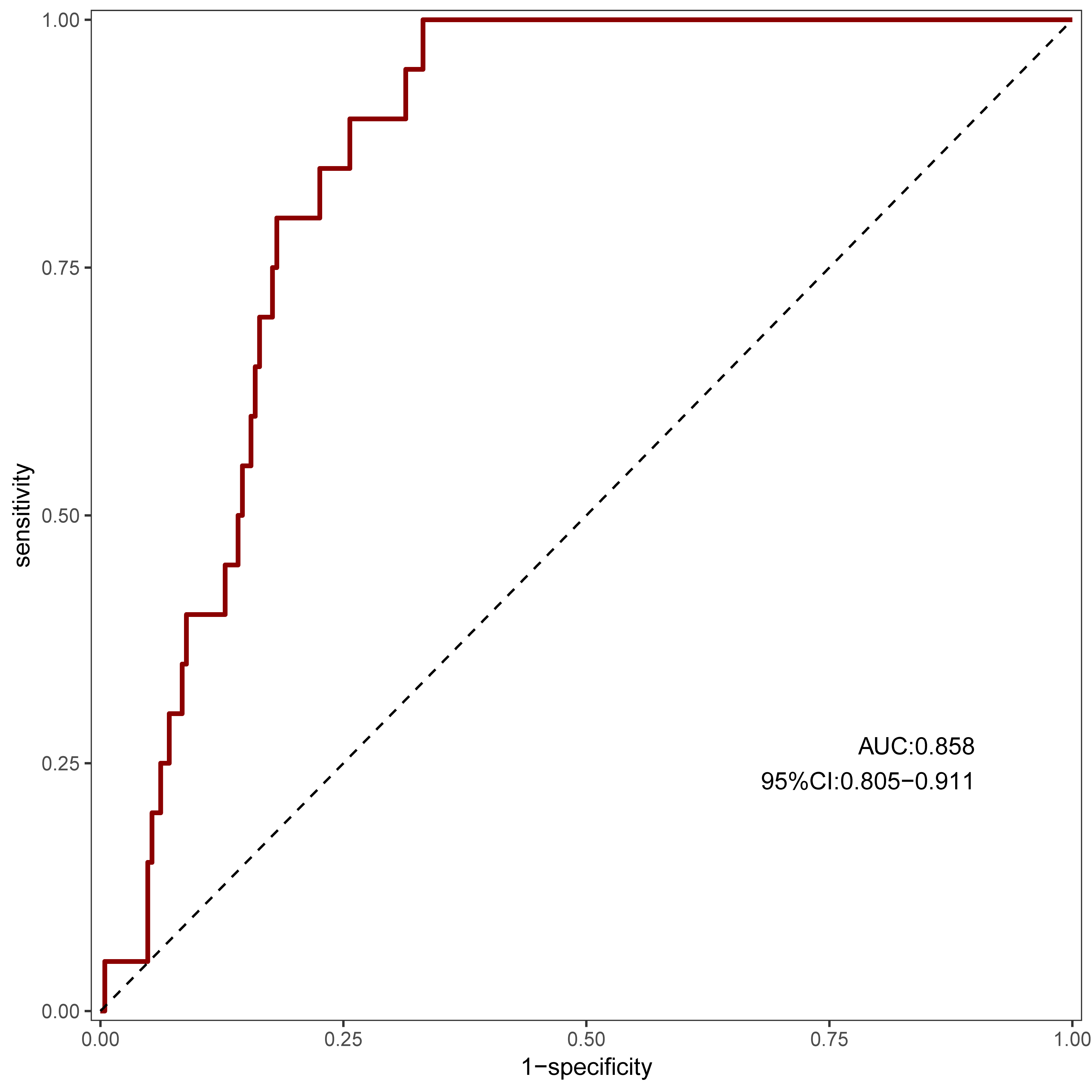

Supplement: Supplementary file 1 [file cimb-47-00208-s001.zip › Supplementary Figure S10.tif]

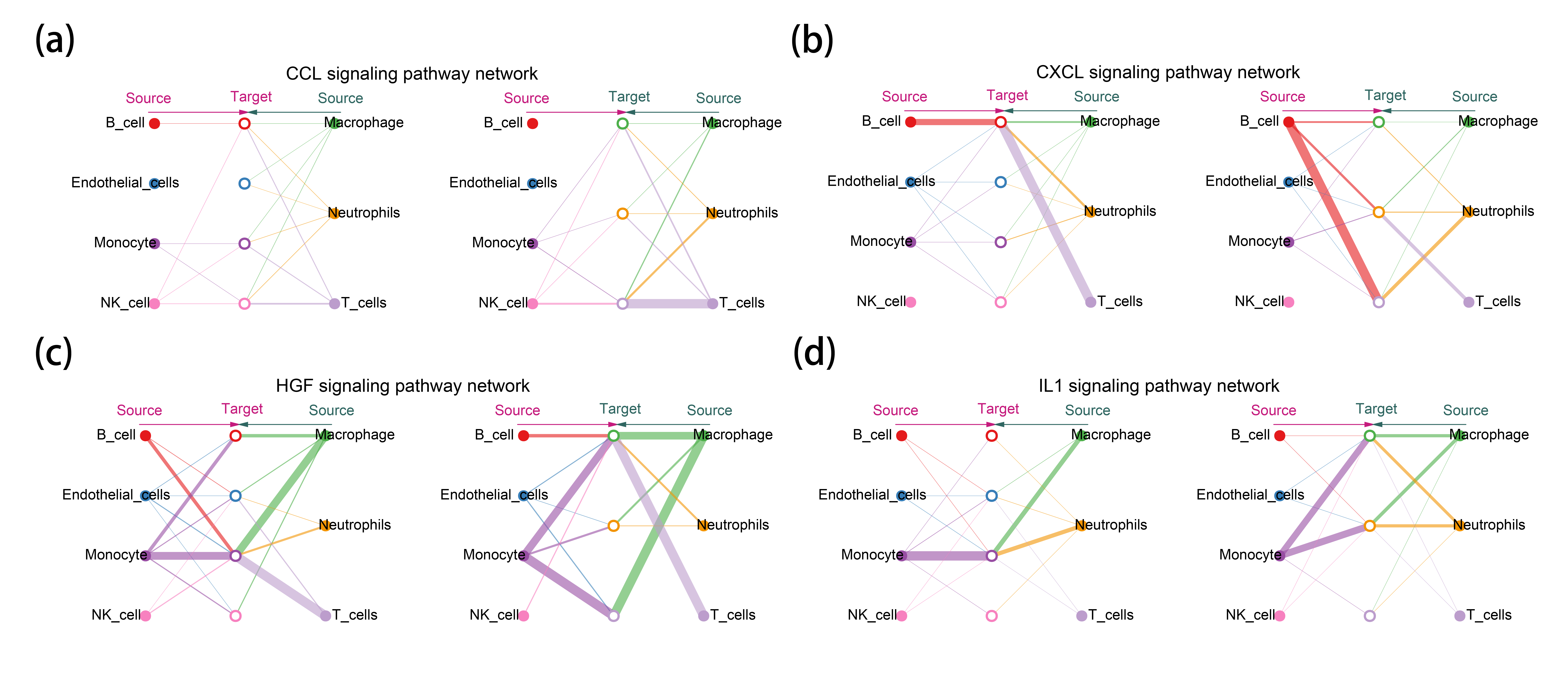

Supplement: Supplementary file 1 [file cimb-47-00208-s001.zip › Supplementary Figure S8.tif]

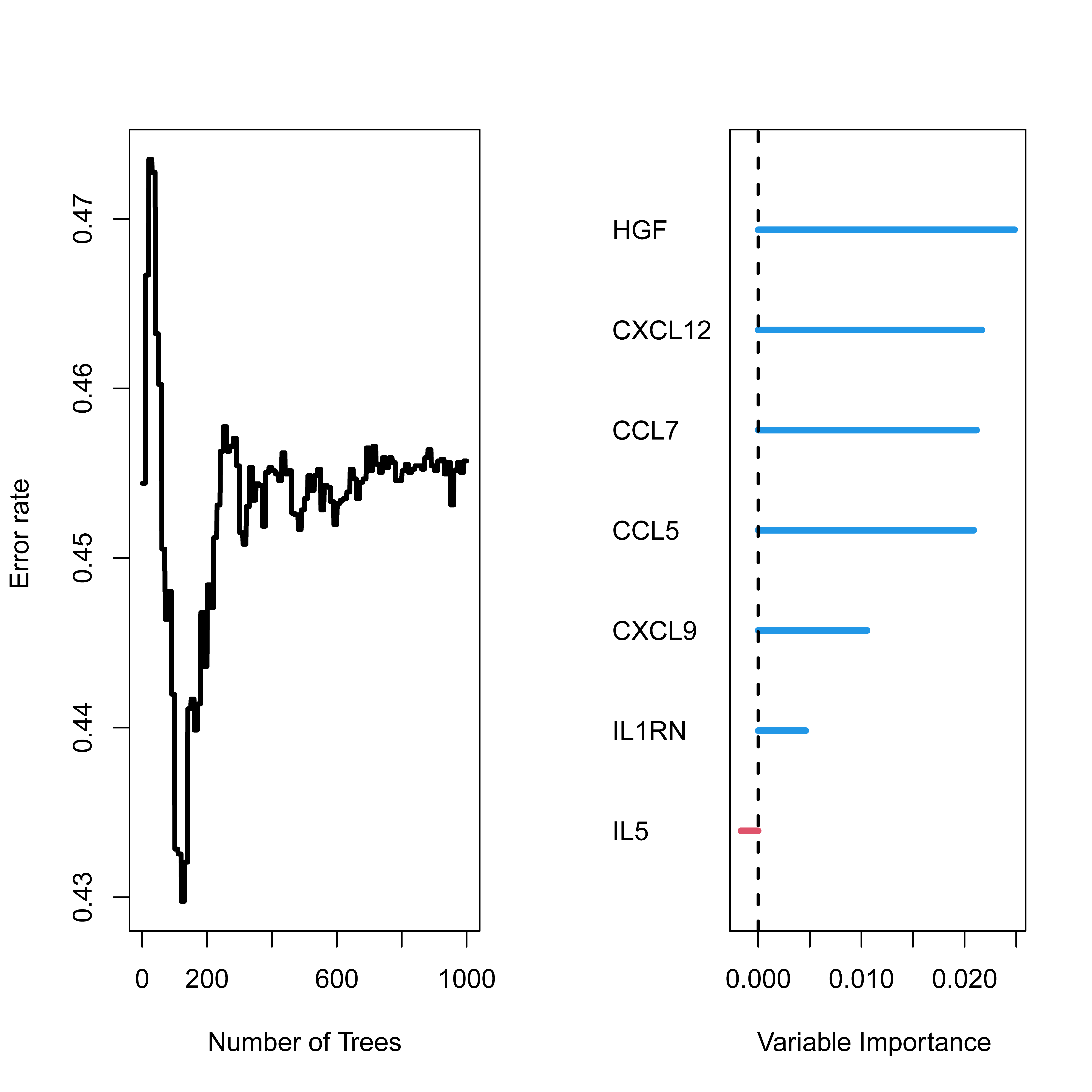

Supplement: Supplementary file 1 [file cimb-47-00208-s001.zip › Supplementary Figure S9.tif]
